# Protection induced by virus-like particle vaccine containing tandem repeat gene of respiratory syncytial virus G protein

**DOI:** 10.1371/journal.pone.0191277

**Published:** 2018-01-16

**Authors:** Ah-Ra Kim, Dong-Hun Lee, Su-Hwa Lee, Ilaria Rubino, Hyo-Jick Choi, Fu-Shi Quan

**Affiliations:** 1 Department of Biomedical Science, Graduate School, Kyung Hee University, Seoul, Korea; 2 Department of Medical Zoology, Kyung Hee University School of Medicine, Seoul, Korea; 3 Department of Chemical and Materials Engineering, University of Alberta, Edmonton, AB T6G 1H9, Canada; University of Iowa, UNITED STATES

## Abstract

Respiratory syncytial virus (RSV) is the leading cause of lower respiratory tract illness in infants, young children and the elderly. However, there is no licensed vaccine available against RSV infection. In this study, we generated virus-like particle (VLP) vaccine and investigated the vaccine efficacy in a mouse model. For VLP vaccines, tandem gene (1–780 bp) for V1 VLPs and tandem repeat gene (repeated 450–780 bp) for V5 VLPs were constructed in pFastBac^TM^ vectors, respectively. Influenza matrix protein 1 (M1) was used as a core protein in the VLPs. Notably, upon challenge infection, significantly lower virus loads were measured in the lung of mice immunized with V1 or V5 VLPs compared to those of naïve mice and formalin-inactivated RSV immunized control mice. In particular, V5 VLPs immunization showed significantly lower virus titers than V1 VLPs immunization. Furthermore, V5 VLPs immunization elicited increased memory B cells responses in the spleen. These results indicated that V5 VLP vaccine containing tandem repeat gene protein provided better protection than V1 VLPs with significantly decreased inflammation in the lungs. Thus, V5 VLPs could be a potential vaccine candidate against RSV.

## Introduction

Respiratory syncytial virus (RSV) is responsible for the majority of lower respiratory tract infections in infants, young children and the elderly, as well as in immunocompromised hosts and individuals suffering from chronic lung disease worldwide [1–3]. In the 1960s, a vaccine trial with formalin-inactivated RSV (FI-RSV) failed to protect against RSV and resulted in severe vaccine enhanced respiratory disease (ERD) upon RSV infection [4]. Despite extensive attempts, there is no licensed vaccine against RSV infection.

RSV is a member of the *Pneumovirus* genus within the *Paramyxoviridae* family of enveloped, single-stranded, negative-sense viruses. The viral genome encodes 10 mRNAs, which produce 11 viral proteins. Glycosylated G and fusion F proteins are the two major surface glycoproteins of RSV, and they induce protection against RSV [5–7]. A previous study has demonstrated that a region of the RSV G protein located between amino acids 184 and 203 is involved in protective immunity against RSV [8]. This region induces helper T cell (T_H_ cell) responses and eosinophilia [9]. Five linear B cell epitopes were identified between amino acids 152 and 163, between 165 and 172, between 171 and 187, and between 196 and 204 [10]. Vaccination with layer–by-layer nanoparticles carrying the RSV G protein CX3C motif (182–186 aa) induced protection against RSV infection in mice [11]. Thus, the region where more T or B cell epitopes and CX3C motif were contained (150–260 aa) was used in this study for tandem repeat gene.

Virus-like particles (VLPs) are morphologically similar to live viruses, but lack viral genetic materials, and therefore cannot replicate. VLPs have shown promising results in preclinical and clinical studies in terms of both safety and efficacy [12]. In our previous study, we found that VLP vaccines containing RSV G glycoprotein induced superior protection than those containing fusion protein [13]. Thus, we searched the region for tandem repeat in glycoprotein G where more T or B cell epitopes exist.

In this study, we developed VLP vaccines V1 and V5 where V1 plasmid contains the G (bp 1–780) and V5 plasmid G (bp 1–780) plus G (bp 450–780) in tandem. Influenza M1 protein was used as a core protein in generating V1 and V5 VLPs [13]. We hypothesized that VLPs containing tandem repeat gene will induce strong T or B cell immune response and protection. We compared immune response, inflammation and protection against RSV A2 virus challenge induced by V1 and V5 VLP vaccines in a mouse model.

## Materials and methods

### Ethics statement

All animal experiments and husbandry involved in these studies were conducted under the guidelines of the Kyung Hee University IACUC. All animal procedures performed in this study were reviewed, approved, and supervised by an animal research ethics committee in Kyung University (permit number: KHUASP (SE) - 16–009). The researchers were trained in animal care and handling, and received the certificate of completion for Animal Welfare & Ethics Course from the CITI.

### Cells, viruses and antibodies

*Spodoptera frugiperda* SF9 insect cells were maintained in suspension in serum-free SF900Ⅱ medium (Invitrogen) at 27°C. HEp-2 cells were obtained from ATCC. HEp-2 cells were grown in tissue culture flasks in Dulbecco’s modified Eagle medium (DMEM) with 10% fetal bovine serum (FBS), penicillin and streptomycin at 37°C with 5% CO_2_. Respiratory syncytial virus A2 (RSV A2) was originally kindly provided by Dr. Marty Moore (Emory University). MDCK cells were infected with influenza virus (A/PR/8/34) to obtain influenza virus total RNA. Monoclonal mouse anti-RSV fusion protein (131-2A) was obtained from Millipore and used in virus plaque assay. Mouse monoclonal antibody to influenza A virus M1 was obtained from Abcam and used in western blot. HRP-conjugated goat anti-mouse immunoglobulins G (IgG), IgG1 and IgG2a, were obtained from Southern Biotech and used as secondary antibodies.

### Preparation of RSV A2 and FI-RSV

HEp-2 cells were grown in tissue culture flasks in DMEM containing 10% FBS, penicillin and streptomycin. RSV A2 in serum-free DMEM was added to HEp-2 cells and adsorbed for 1 h at 37°C with 5% CO_2_. DMEM with 10% FBS was added to the flask and incubated at 37°C for 2–3 days. RSV-infected cells were harvested using a cell scraper and centrifuged at 3000 rpm for 30 min at 4°C to remove the supernatants. Infected cell pellets were sonicated and centrifuged at 4°C, and the supernatants were titrated by immune-plaque assay as described previously [13], and stored at -80°C. FI-RSV was prepared by modification of the previously described method [14,15]. Briefly, RSV stocks were inactivated with formalin (1:4000 v/v) for 3 days at 37°C. The formalin-treated virus was pelleted by ultracentrifugation at 17,700 g for 17 h at 4°C. The formalin inactivated pellet was resuspended in serum-free medium with addition of 4 mg/ml aluminum phosphate, and then sonicated for 15 sec and stored at 4°C.

### Construction of recombinant baculovirus (rBV) expressing tandem repeat gene of RSV G or influenza M1

Total RNA was extracted from the RSV (subtype A, strain A2)—infected HEp-2 cells using RNeasy Mini Kit (Qiagen) and complementary DNA (cDNA) was synthesized. Tandem (1’ tandem) of RSV G gene was amplified by polymerase chain reaction (PCR) from cDNA with forward primer 5’-AAAGAATTCATGTCCAAAAACAAGGACCAAC-3’ and reverse primer 5’-TTAGTCGACGGCGGCTGTGAGTTCTGGATTTCC-3’ (*Eco*R and I *Sal I* enzyme restriction sites were underlined). Tandem (2’ tandem) was also amplified by PCR from cDNA with forward primer 5’-AAAGTCGACGGCGGCCAACGCCAAAACAAACCA-3’ and reverse primer 5’-TTATCTAGAGGCGGCTGTGAGTTCTGGATTTCC-3’ (*Sal I* and *Xba I* enzyme restriction sites were underlined). The transmembrane (TM) and cytoplasmic tail (CT) regions from the influenza A virus PR8 hemagglutinin (HA) were generated as described previously [16]. For tandem repeat generation, 4 × Gly linker (GGCGGC) was inserted in reverse primers for 1’ tandem and 2’ tandem. For influenza M1 gene cloning, MDCK cells were infected with A/PR/8/34 virus, total RNA was extracted using an RNeasy Mini kit (Qiagen), and then cDNA was synthesized. Influenza M1 gene was amplified by PCR from cDNA with forward primer 5’-TCCCCCGGGCCACCATGAGCCTTCTGACCGAGGTC-3’ and reverse primer 5’-TTACTTCTAGATTACTTGAACCGTTG CATCTG-3’ (*Sma*I and *Xba*I enzyme restriction sites were underlined). PCR products for 1’ tandem, 2’ tandem, HA-TM-CT and influenza M1 were cloned into the pFastBac^TM^ vector (Invitrogen). Tandem (1’tandem) and tandem repeat (1’ tandem + 2’ tandem) of RSV G were fused with HA-TM-CT by ligations as previously described [16]. All clones were confirmed by DNA sequencing (RSV accession number: KU716110; M1 accession number: EF467824), and recombinant plasmid DNAs were stored at -20°C until used.

### Generation of rBV and virus-like particles (VLPs)

Generation of recombinant baculovirus (rBV) was obtained using a Bac-to-Bac expression system (Invitrogen) according to the manufacturer’s instructions. Briefly, DNA plasmids containing tandem of RSV G genes or tandem repeat of RSV G genes were transfected into SF9 cells using cellfectinⅡ (Invitrogen). V1 VLPs were produced by co-infecting SF9 cells with rBV expressing 1’ tandem of RSV G and influenza matrix 1 (M1). V5 VLPs were produced by co-infecting SF9 cells with rBV expressing 1’+2’ tandem of RSV G and M1. To collect the supernatants, cell culture was centrifuged at 6000 rpm for 20 min at 4°C on days 2–3 post-infection. The supernatant was pelleted using ultra-centrifugation. The pelleted VLPs were purified by 20%-30%-60% sucrose gradient at 30,000 rpm for 1 h at 4°C. The VLP bands between 30% and 60% were collected and pelleted at 28000 rpm for 40 min at 4°C. To resuspend the VLPs, they were incubated in PBS overnight at 4°C. Protein concentration was determined using BCA Assay Kit (Sigma Aldrich).

### Characterization of VLPs

Western blots and electron microscopy were used to characterize the VLPs. For western blot analysis, mouse serum that was infected with RSV A2 twice at an interval of 4 weeks was used to probe single tandem and repeat tandems of RSV G protein. For M1 protein detection, monoclonal mouse anti-M1 antibody was used. HRP-conjugated goat anti-mouse IgG was used as secondary antibody. VLPs were negatively stained using phosphotungstic acid (pH 7.0) and transmission electron microscopy (TEM; JEOL 2100, JEOL USA, Inc.; Peabody, MA, USA) was performed at 200 kV to characterize the VLP morphology [17].

### Mice immunization, challenge and sample collection

Female BALB/c mice aged 7 weeks were immunized intranasally twice with V1 VLPs (25 μg/mouse, total VLP protein) and V5 VLPs (25 μg/mouse, total VLP protein) at 4 weeks interval. For FI-RSV immunization, 50 μL of FI-RSV was inoculated once by intranasal route. Each group contained 6 mice. Blood was sampled by puncture of the retro-orbital plexus at 1 week and 4 weeks after prime and boost administration. Serum samples were collected and stored at -20°C. At 4 weeks after boost immunization, naïve or immunized mice were challenge infected with 4.4 × 10^6^ PFU/50 μL of RSV A2 under isoflurane anesthesia. Mice were sacrificed by cervical dislocation after anesthesia under the guidelines of the IACUC at day 4 post-challenge. Individual organs including lung and spleen were collected at day 4. The experiments were performed three times. All animal experiments and husbandry involved in these studies were conducted under the guidelines of the Kyung Hee University IACUC.

### Antibody response

RSV A2 specific antibodies (IgG, IgG1 and IgG2a) were determined in sera by enzyme-linked immunosorbent assay (ELISA). Briefly, 96-well plates were coated with 4 μg/mL of RSV A2 as an ELISA antigen at 4°C overnight. The plates were washed, and then blocked with 0.2% gelatin in PBST for 2 h at 37°C. After washing, diluted sera were added and incubated for 2 h at 37°C. Antibody responses were detected using the HRP-conjugated goat anti-mouse IgG, IgG1 and IgG2a secondary antibodies. The substrate *O*-phenylenediamine in citrate-phosphate buffer (pH 5.0) containing 0.03% H_2_O_2_ was used to develop color and stopped with 2N H_2_SO_4_. The optical density at 490 nm was measured with an ELISA reader.

### Flow cytometry analysis

To perform cell phenotype analysis, single cell suspensions from lung and spleen were isolated from homogenized tissues. 1 × 10^6^ splenocytes and lung leukocytes were stained with surface marker antibodies including CD45-PerCP, CD11b-APC, CD23-FITC, F4/80-PE, CD11c-FITC, SiglecF-PE, B220-FITC, CD27-PE-Cy7 and IgG1-PE (BD Biosciences). Stained cells were acquired on a BD FACSCalibur and BD AcuriC6 and analyzed using Flow Jo software and BD AcuriC6 software.

### Lung histopathology

Individual lung lobes harvested from mice at day 4 post-challenge were inflated and fixed with 10% neutral buffered formalin. The lung tissues (n = 6) were embedded in paraffin, sectioned and stained with hematoxylin and eosin (H&E) and periodic acid-schiff (PAS) stains to assess histologic changes, eosinophil and mucin expression. Tissue sections stained with H&E were scored blindly for degree of eosinophilia on a scale of 0 to 5 (0 = absent and 5 = severe). For sections stained with PAS, degree of mucin production was determined on a scale of 0 to 5 (0 = absent and 5 = severe). At least eight sections per mouse were obtained for histopathologic analysis. Cell counts from bronchoalveolar lavage (BAL) were performed to determine lung inflammation. Six mice from each experiment were used to count BAL cell number as described [18].

### Lung virus titer by RSV immune-plaque assay

Viral titers in individual lung homogenates were quantified at day 4 post-challenge by immune-plaque assay. Lung tissues were homogenized with 1 mL of PBS using a syringe, and filtered through 100 μm pore size cell strainers. The lung virus load was determined by plaque assay on HEp-2 cell. Briefly, confluent HEp-2 cells were grown in 24-well plates. Following serial dilution with DMEM media without FBS, lung homogenates from infected mice were added to the plates, incubated for 1 h at 37°C and removed. 1 mL of overlay was added to each well, and the plates were incubated for 2 days at 37°C. Overlay was removed and ice-cold acetone-methanol (1:1) was used for 10 min to fix the cells. After washing, Hep-2 cells in wells were blocked with 5% skim milk in PBS for 30 min. Monoclonal mouse anti-RSV fusion protein and HRP-conjugated goat anti-mouse IgG in PBS were used as primary and secondary antibody, respectively. Each antibody was incubated for 1 h at 37°C. DAB substrate was used to develop individual plaques.

### Statistics

All parameters were recorded for individuals within groups. Statistical comparisons of data were carried out using One-way ANOVA of PC-SAS 9.3. A P value < 0.05 was considered to be significant.

## Results

### Constructs generation

**Fig 1:**
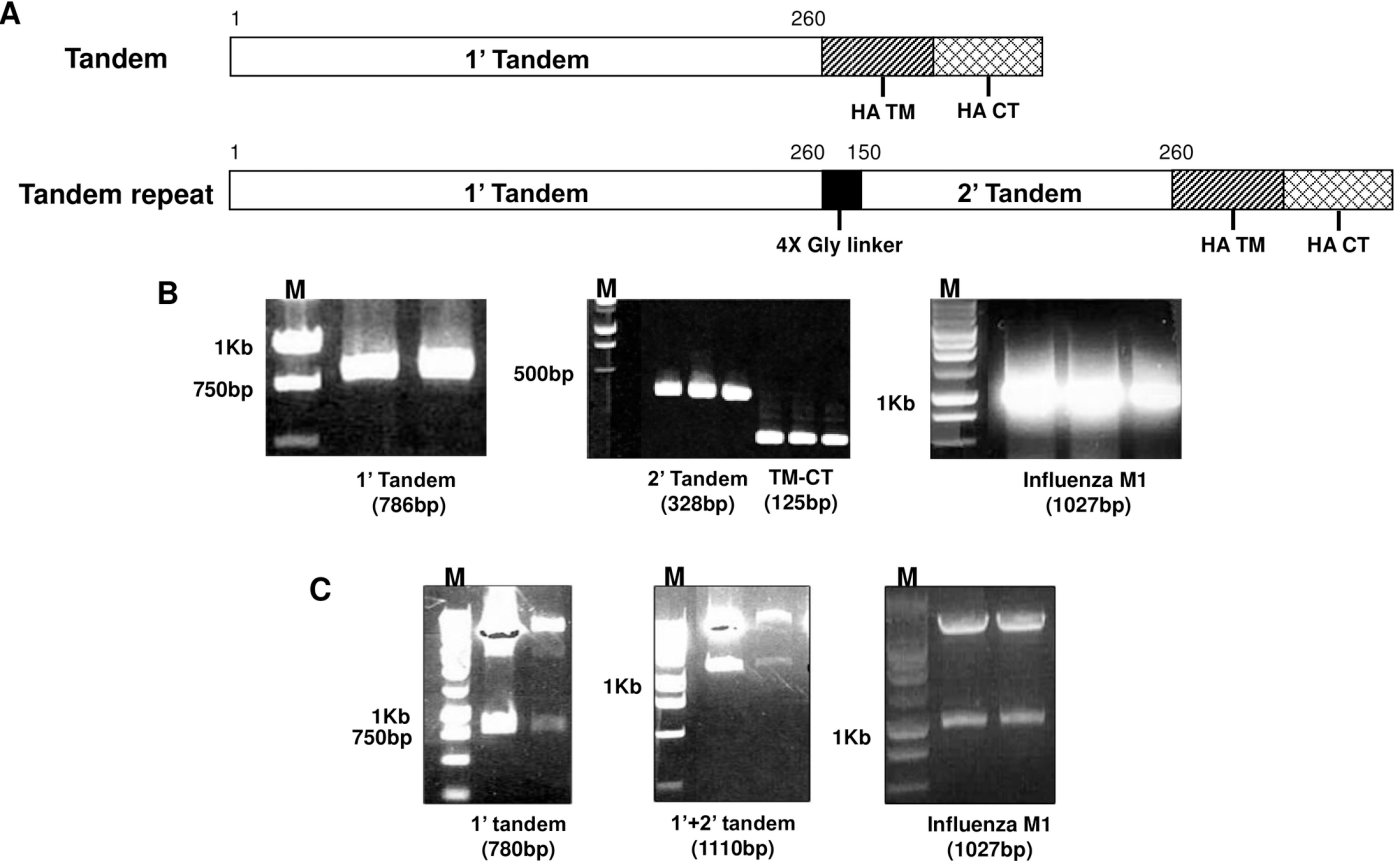
Composition, amplification and gene cloning. Tandem repeat gene of RSV G glycoprotein was designed as indicated (A). For V1 VLPs, 1’ tandem includes between 1 and 260 amino acids of G glycoprotein from start codon. For V5 VLPs, tandem repeat consisting of 1’ tandem and 2’ tandem includes between 150 and 260 amino acids of G glycoprotein. PCR products for 1’, 2’ tandem, HA-TM-CT and influenza M1 were amplified, respectively. (B). Genes were cloned into pFastBac^TM^ vectors for baculovirus production (C).

Constructs for tandem repeat gene study were designed as indicated in Fig 1A. Single tandem gene contained sequence from 1 to 780 base pair (260 amino acid) of RSV G glycoprotein. Tandem repeat gene contained 1’ tandem and 2’ tandem as indicated, consisting of repeat genes from 450 to 780 base pair (150–260 amino acids) of RSV G glycoprotein. PCR products for clones were amplified (Fig 1B), and tandem (1’ tandem), tandem repeat (1’+2’ tandem) and influenza M1 genes were cloned into pFastBac^TM^ vectors (Fig 1C).

### Production and characterization of VLPs

**Fig 2:**
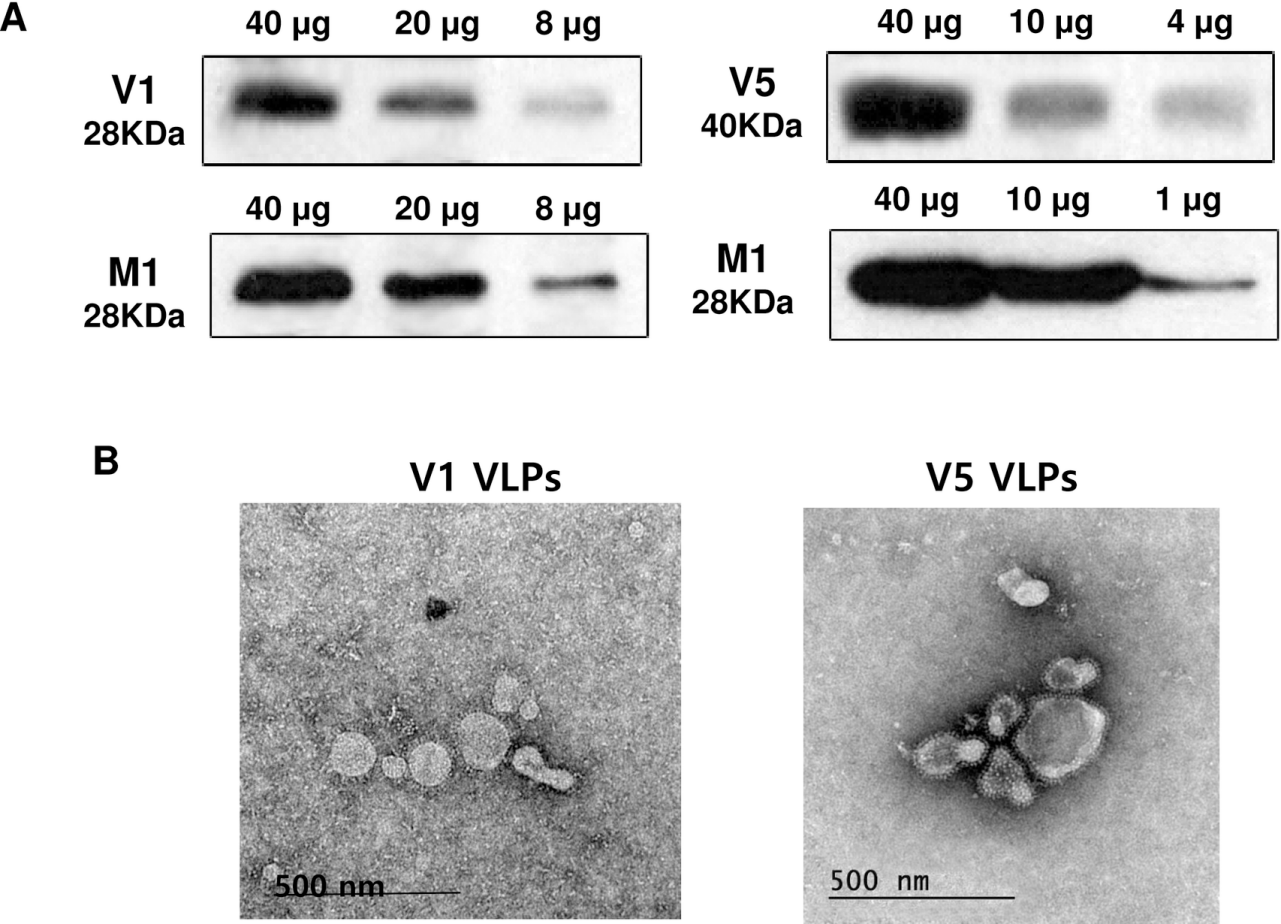
Characterization of virus-like particles (VLPs). V1 and V5 VLPs expressing 1’ tandem or 1’ + 2’ tandem together with influenza M1 were produced. V1 and V5 VLPs were characterized by western blots (A) and electron microscopy. V1 or V5 VLPs were indicated by arrows (B). The molecular weights of 1’ tandem, 1’ + 2’ tandem and M1 were 28 KDa, 40 KDa and 28 KDa, respectively. The spikes representing tandem from V1 VLPs or tandem repeat form V5 VLPs were observed on the surface of VLPs.

V1 VLPs (tandem) and V5 VLPs (tandem repeat) were produced by co-infected 1’ tandem or 1’ + 2’ tandem with influenza M1 recombinant baculoviruses. The components of generated V1 VLPs and V5 VLPs were confirmed by western blot (Fig 2A). V1 and V5 VLPs were reacted with mouse sera that was infected with RSV A2 (28 kDa and 40 kDa), respectively. Influenza M1, used as VLPs core protein, was detected by monoclonal mouse anti-M1 antibody (28 kDa). V1 and V5 VLPs exhibited spherical shapes under microscopy and spikes were observed on the surface of the VLPs (Fig 2B).

### VLPs immunization induced humoral immunity

**Fig 3:**
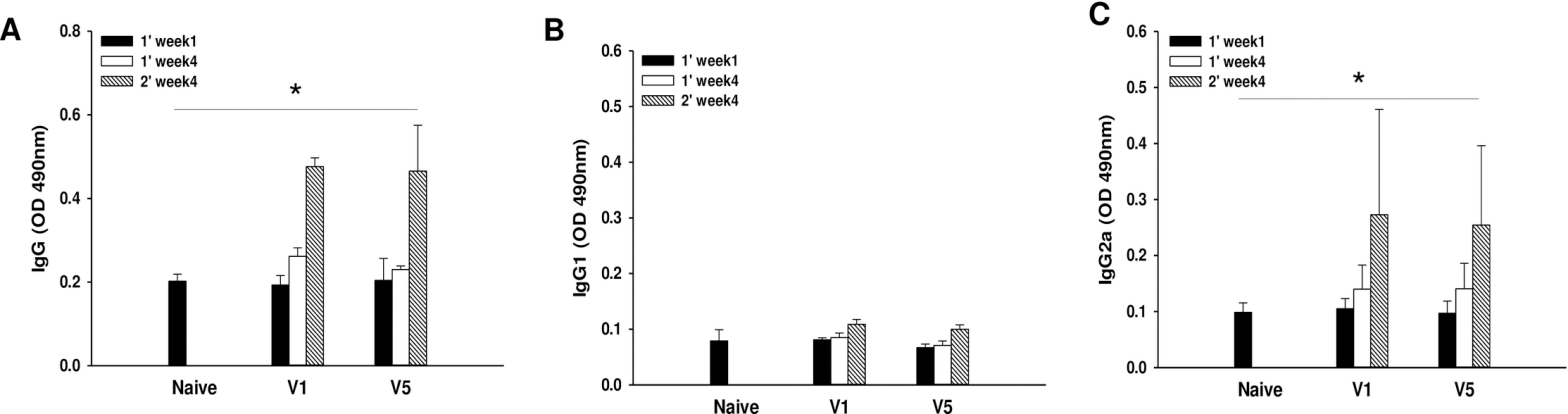
RSV A2-specific antibody response and memory B cell phenotyping. RSV A2-specific antibody responses were determined as described previously [13]. Total IgG, IgG1 and IgG2a levels were determined in the sera (n = 6) at weeks 1 and 4 from primary (1’week1) and at week 4 from secondary immunization (2’week4). IgG levels were significantly increased in the immunized groups (A, *P < 0.05)). Higher levels of IgG2a antibody responses were observed in immunized mice (C, *P < 0.05)) compared to that of IgG1 antibody responses (B). The data shown are representative of three independent experiments.

**Fig 4:**
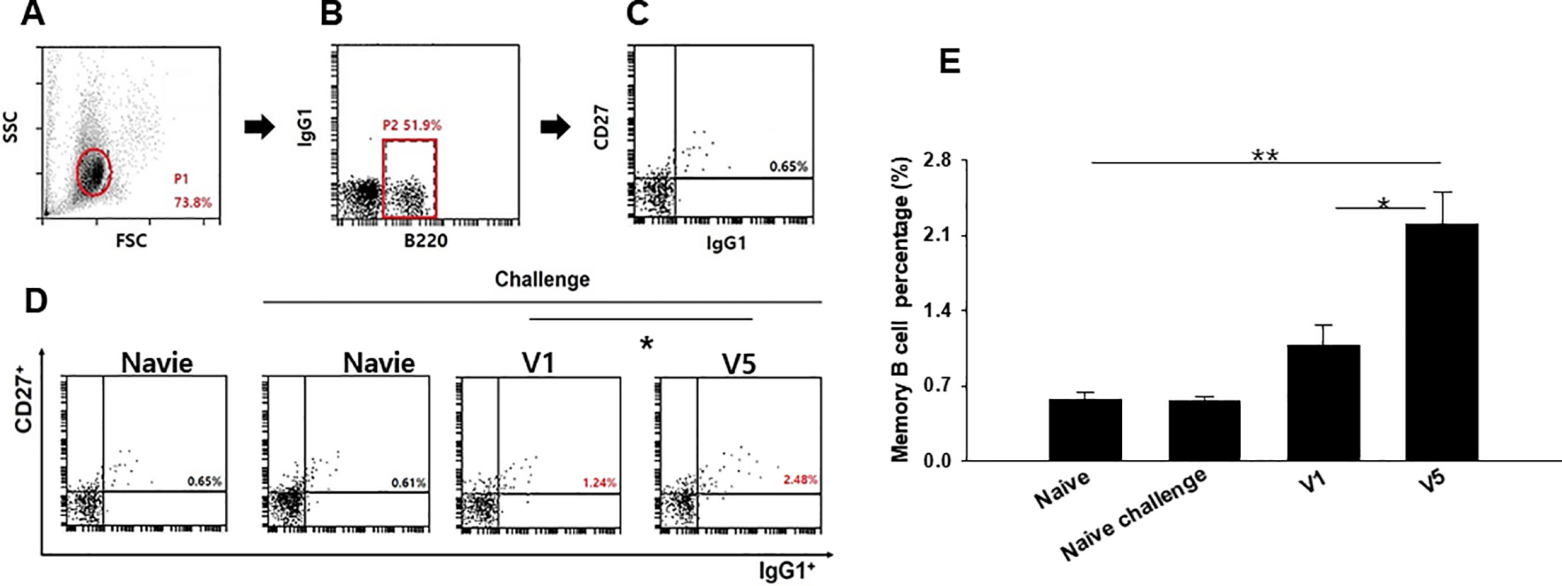
Memory B cell response. To investigate the levels of memory B cells in the spleen, the isolated cells from the spleen (n = 6) were stained with B220, CD27 and IgG1. Fig 4A–4C showed the gating strategy for the plots. Memory B cells (CD27+IgG1+) in B220+ population were measured (D), and V5 VLPs immunized mice showed a higher percentage of memory B cells (D, E, *P < 0.05, **P < 0.01). The data shown are representative of three independent experiments.

To evaluate RSV A2-specific serum antibody induced by V1 VLPs or V5 VLPs immunization, the total IgG, IgG1 and IgG2a antibody levels were determined at week 1 and 4 after prime and week 4 after boost immunization. Significantly higher levels of RSV A2-specific IgG and IgG2a responses from mice immunized with V1 or V5 VLPs were observed after boost compared to after prime immunization (Fig 3A and 3C, *P < 0.05). IgG1 antibodies were not detected in serum samples, while a strong IgG2a antibody response was observed (Fig 3B and 3C). There is no significant difference of IgG2a between the V1 and V5 VLPs. These results indicate strong immunogenicity of both V1 VLPs and V5 VLPs, with induction of IgG2a dominant antibody responses. Memory B cell response was determined in the spleen by flow cytometry [19]. Fig 4A–4C show the gating strategy for the plots. As shown in Fig 4D, V1 VLPs or V5 VLPs immunization showed increased population of memory B cells compared to those from naïve or naïve challenge. In particular, V5 VLPs immunization (2.27%) showed significantly increased memory B cells compared to V1 VLPs immunization (1.13%) (Fig 4D and 4E, *P < 0.05). The greater memory B cell response to V5 VLPs immunization as compared to V1 VLPs immunization might contribute to better protection in mice immunized with V5 VLPs.

### V5 VLPs immunization significantly reduced lung inflammation upon RSV challenge infection

**Fig 5:**
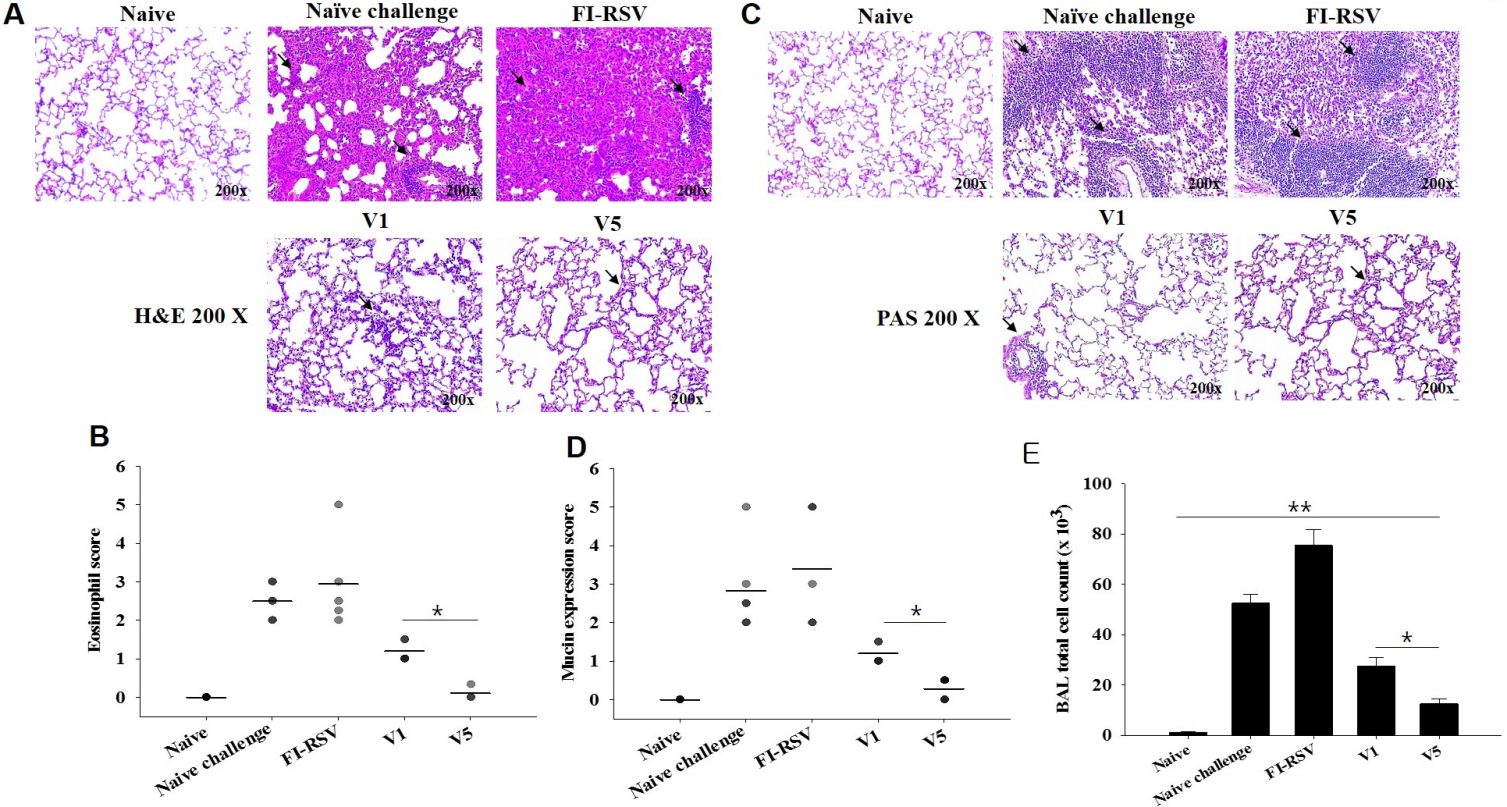
Lung eosinophil and mucin expression observed by histopathology. Lung tissues (n = 6 mice) were collected from individual mice on day 4 after challenge, and tissue sections were stained with Hematoxylin and eosin (H&E) and Periodic acid-Schiff (PAS) to assess pulmonary histopathologic changes. Eosinophils of H&E staining (A) and score (B, *P < 0.05)) for eosinophil response were determined. PAS staining (C) and scores (D, **P < 0.005)) for mucin production were determined. FI-RSV immune mice showed severe inflammation and mucin production, whereas mice immunized with VLPs showed no inflammation and no mucin production upon challenge. BAL cell counts were performed at day 4 post-challenge infection from mice immunized with V1 or V5 VLPs and from FI-RSV immunized mice (E: *<0.05, ** < 0.005).

**Fig 6:**
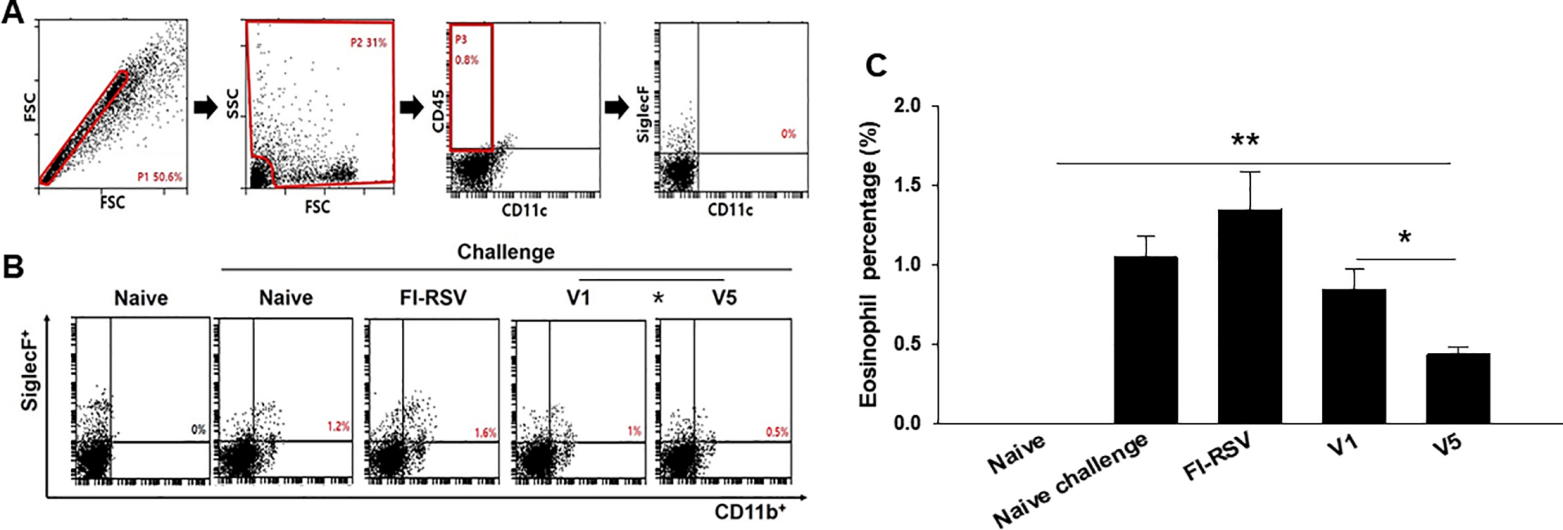
Eosinophil response in the lung observed by cell phenotyping. To understand the eosinophil cell phenotypes in the lung, cells from the lung (n = 6) were isolated and stained with surface markers. Eosinophil (CD11b^+^SiglecF^+^) in CD45^+^CD11c^–^ total cells were analyzed (A, B and C, *P < 0.05, **P < 0.01). The data shown are representative of three independent experiment. FI-RSV immunized mice showed significantly high levels of eosinophil and macrophage response compared to naïve or naïve challenge control mice. In contrast, V1 and V5 VLPs immunized mice showed lower eosinophil responses compared to FI-RSV and naïve challenge control. Compared to V1 VLPs, V5 VLPs showed lower eosinophil responses. The data shown are representative of three independent experiments.

**Fig 7:**
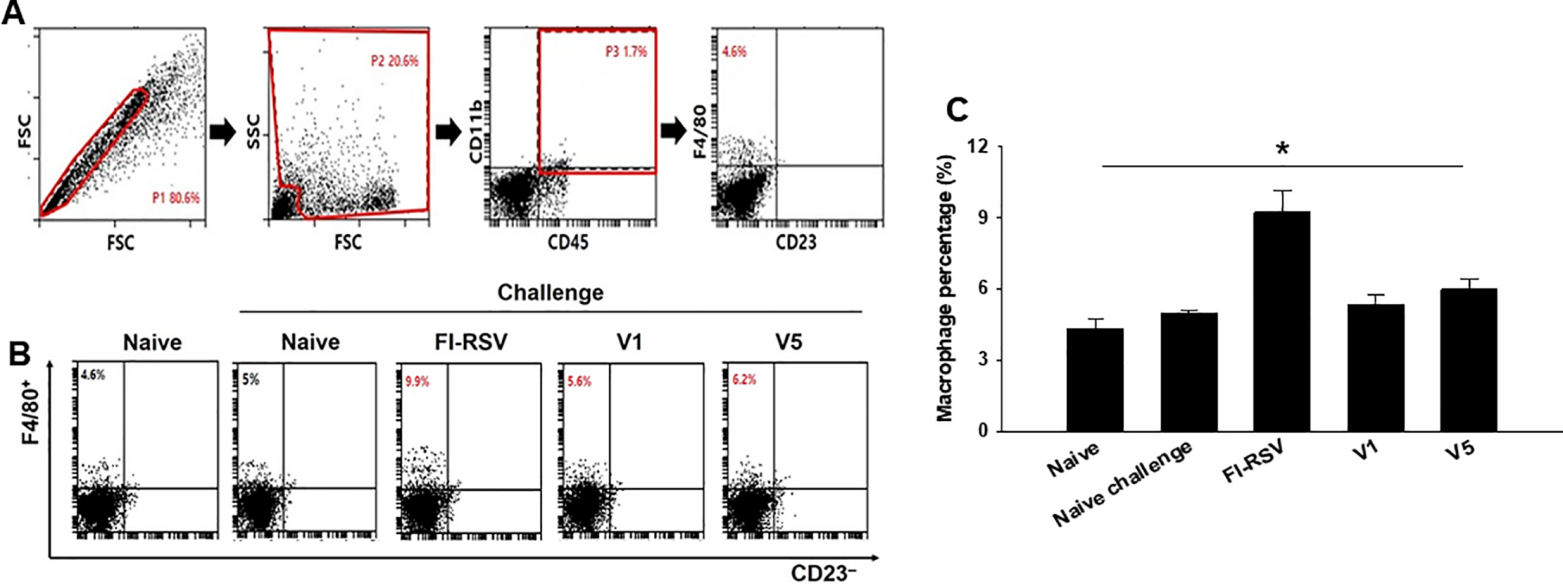
Macrophage response in the lung observed by cell phenotyping. To understand the macrophage populations in the lung, cells from the lung (n = 6) were isolated and stained with surface markers. Macrophage phenotype gated on F4/80+CD23– in CD45+CD11b+ total cells was observed (Fig 7A, 7B and 7C, *P < 0.05). Fig 7A showed the gating strategy for the plots. V1 and V5 VLPs showed similar levels of macrophage infiltration to naïve or naïve challenge control upon RSV A2 challenge infection. The data shown are representative of three independent experiments.

Pulmonary histopathologic changes upon RSV infection were assessed to evaluate the safety of RSV vaccine. Eosinophils infiltration from lung tissues was examined using H&E staining (Fig 5A) and scored (Fig 5B, *P < 0.05). FI-RSV immunized mice showed a more massive influx of eosinophils than V1 or V5 VLPs immunized mice. In particular, V5 VLPs immunized mice showed significantly lower eosinophils infiltration score compared to V1 VLPs immunized mice (Fig 5A and 5B, *P < 0.05). Concurrently, mucin production was investigated using PAS staining (Fig 5C) and scored (Fig 5D, *P < 0.05). Excessive mucin production was observed in FI-RSV immunized mice. In contrast, mucin production was not observed in mice immunized with V1 or V5 VLPs. BAL cell counts from each mouse were performed to determine pulmonary inflammation. As indicated in Fig 5E, significantly reduced BAL cells were found in V1 or V5 immunized mice compared to FI-RSV or naïve challenge groups (**P<0.005). V5 VLPs immunized mice showed significantly lower numbers of BAL cells compared to V1 VLPs (*P<0.01). These results indicate that V1 or V5 VLPs immunization significantly reduced lung inflammation upon RSV A2 live virus challenge infection. Flow cytometry analysis was used to assess lung eosinophil and macrophage infiltration (Fig 6, Fig 7). In contrast to the high levels of eosinophils (CD11b^+^SiglecF^+^ in CD45^+^CD11c^–^ total cells) shown in FI-RSV immunized mice (1.5%), V1 or V5 VLPs immunized mice exhibited lower levels of eosinophils than naïve challenge control (0.9% or 0.4% vs 1.1%). Notably, V5 VLPs immunized mice showed significantly lower levels of eosinophils (0.4%) compared to V1 VLPs immunized mice (0.9%) (Fig 6, P < 0.05). To investigate the macrophage infiltration in the lung, lung cells were isolated from mice at day 4 post-challenge and stained with surface markers. Macrophage phenotype gated on F4/80^+^CD23^–^ in CD45^+^CD11b^+^ total cells was observed (Fig 7). Fig 7A indicates the gating strategy for the plots. As shown in Fig 7B, the highest levels of macrophages were observed in FI-RSV immunized mice (9.9%) upon RSV infection. In contrast, V1 VLPs (5.6%) or V5 VLPs (6.2%) immunized mice showed similar levels to naïve control (5.0%). These results indicate that V1 or V5 VLPs immunization significantly reduced lung inflammation upon RSV A2 challenge infection.

### VLPs immunization significantly reduced lung virus loads upon RSV challenge infection

**Fig 8:**
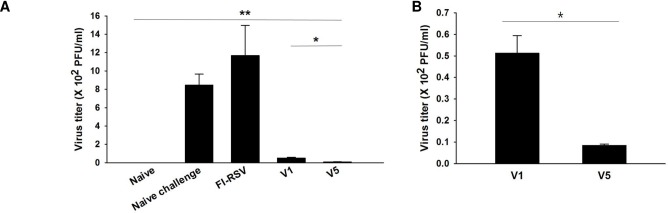
Lung viral titers upon RSV A2 challenge infection. Lung viral titers were determined from mice immunized with V1 or V5 VLPs and from FI-RSV immunized mice. Lung tissues (n = 6) were collected from individual mice at day 4 after challenge. Mice immunized with V1 or V5 VLPs showed significantly lower levels of viral titers compared to naïve control and FI-RSV immunized mice (A, **P < 0.005). Additionally, V5 VLPs showed significantly lower virus titers compared to V1 VLPs (B, *P < 0.05).

Lung virus loads following infection are the most prominent indicator to assess vaccine protective efficacy. Immunized and naïve mice were infected with RSV A2 virus (4.4 × 10^6^ PFU/mouse) at 4 weeks after boost, and lung virus titers were determined at day 4 post-infection. As shown in Fig 8, significantly decreased lung virus loads were detected in V1 or V5 VLPs immunized mice compared to FI-RSV immunized and naïve mice (**P < 0.005). Furthermore, lower lung virus loads were found in V5 VLPs immunized mice than in V1 VLPs immunized mice (5-fold reduction in virus titer, B: *P < 0.05). These results indicate that V1 or V5 VLPs immunization effectively inhibited virus replication in the lung, and V5 VLPs immunization containing tandem repeat elicited better protection than V1 VLPs immunization.

## Discussion

In this study, we investigated the protective efficacy of VLPs containing a single tandem (V1 VLPs) or tandem repeat (V5 VLPs) from RSV G glycoprotein against RSV A2 infection in a mouse model. We found that V5 VLPs showed significantly decreased lung inflammatory responses and lung virus load compared to V1 VLPs, resulting in better protection in mice immunized with V5 VLPs than with V1 VLPs.

Although different approaches to develop RSV vaccines (subunit vaccines, attenuated viruses, DNA or live vector) have been explored, vaccine protective efficacy has not yet been successful [13]. Thus, alternative strategies to develop RSV vaccine are urgently needed. In the present study, we developed for the first time VLPs containing tandem repeat derived from RSV G glycoprotein, where more T or B cell epitopes exist. In our previous study, we successfully generated RSV VLPs containing RSV G or F proteins and influenza M1 as a core protein [13]. However, lung inflammatory responses upon challenge infection were not assessed [13]. Thus, we focused on determining pulmonary inflammation and protective efficacy in mice immunized with V1 or V5 VLP vaccines. Since RSV G VLP vaccine showed better protection than RSV F VLP [13], we targeted RSV G protein to generate V1 and V5 VLPs. Our data indicated that FI-RSV immunized mice showed severe pulmonary histopathology, including high levels of eosinophil infiltration and mucin production upon RSV infection. In contrast, V1 or V5 VLPs immunized mice showed low inflammatory responses in the lungs. In particular, V5 VLPs showed lower eosinophil infiltration than V1 VLPs, supporting the result that decreased lung inflammation may contribute to lower lung virus loads in mice immunized with V5 VLP vaccine compared to V1 VLPs.

In RSV vaccine development to non-live virus vaccines given to RSV naïve young children, not inducing vaccine-enhanced diseases constitutes the most prominent challenge. Eosinophils accumulation is used as a critical sign against vaccines targeting RSV in lung sections of immunized and challenged BALB/c mice [20,21], since an excess in eosinophils was found in the lungs of infants killed upon immunization with a formalin-inactivated RSV [14]. The V5 VLPs we generated showed significantly reduced eosinophils (0.4%) in lung section compared to V1 VLPs (0.9%), or naïve (1.1%), or FI-RSV (1.5%) mice upon challenge infection. These are encouraging results indicating V5 VLPs containing tandem repeat of RSV G protein could be a potential vaccine candidate against RSV. In a recent study, it has been reported that RSV infection leads to an increase in IL-33 producing alveolar macrophages, inducing Th2 immunity and causing inflammation [22]. Moreover, high levels of macrophages have been reported in FI-RSV immunized mice [23]. In good agreement with the reported findings, we observed an excessive macrophage infiltration in the lung of FI-RSV immunized mice. Conversely, V1 or V5 VLPs immunized mice did not show macrophage infiltration (Fig 7), indicating that VLPs vaccination does not induce an increased presence of alveolar macrophages, and thus exhibits significantly reduced pulmonary inflammation. RSV-G VLPs have been reported to elicit IgG2a dominant, RSV-specific IgG antibody responses against RSV A2 in the sera [13]. Enhanced disease after RSV challenge has been reported to be related to Th2 (IgG1) allergy-like or Th2-associated responses [24–26]. We found that V1 and V5 VLPs induced high levels of IgG2a isotype responses with very low levels of IgG1 isotype responses (Fig 3B and 3C) and lower lung eosinophil infiltration compared to FI-RSV or naïve challenge groups (Fig 5, Fig 6), supporting that Th2 cells contribute to the induction of pulmonary eosinophil in RSV vaccine-enhanced disease [27].

V1 or V5 VLPs vaccination induced significantly high levels of memory B cells (CD27^+^IgG1^+^) in B220^+^ population (Fig 4). Specifically, V5 VLPs (2.27%) showed a remarkably higher memory B cell response than V1 VLPs (1.13%), naïve (0.57%) and naïve challenge (0.50%) groups. This is promising since enhanced memory B cells from VLPs may contribute to protection induced by V1 and V5 VLPs. In fact, memory B cells have a crucial role in generating an accelerated, antibody-mediated immune response. Upon challenge infection, B cell clones increase to generate a polyclonal response and memory B cells persist [28,29].

## Conclusions

To the best of our knowledge, this is the first report demonstrating vaccine efficacy induced by RSV VLPs containing tandem repeat. Both V1 and V5 VLP vaccines provide effective protection against RSV infection. Additionally, V5 VLPs containing tandem repeat show less pulmonary inflammation and lower virus titer than V1 VLPs, indicating that V5 VLPs could be a potential vaccine candidate against RSV.
